# The Role of Oxidative Stress in Skin Disorders Associated with Alcohol Dependency and Antioxidant Therapies

**DOI:** 10.3390/molecules30153111

**Published:** 2025-07-25

**Authors:** Joanna Wróblewska, Anna Długosz, Damian Czarnecki, Wioletta Tomaszewicz, Błażej Błaszak, Joanna Szulc, Weronika Wróblewska

**Affiliations:** 1 Department of Medical Biology and Biochemistry, Faculty of Medicine, Ludwik Rydygier Collegium Medicum in Bydgoszcz, Nicolaus Copernicus University in Toruń, 24 Karłowicza St., 85-092 Bydgoszcz, Poland; 2 Department of Food Industry Technology and Engineering, Faculty of Chemical Technology and Engineering, Bydgoszcz University of Science and Technology, 3 Seminaryjna St., 85-326 Bydgoszcz, Poland; blazej.blaszak@pbs.edu.pl (B.B.); joanna.szulc@pbs.edu.pl (J.S.); 3 Department of Preventive Nursing, Faculty of Health Sciences, Ludwik Rydygier Collegium Medicum in Bydgoszcz, Nicolaus Copernicus University in Toruń, 1 Łukasiewicza St., 85-821 Bydgoszcz, Poland; czarneckidamian@cm.umk.pl; 4 Department of Health Law and Policy, Faculty of Health Sciences, Ludwik Rydygier Collegium Medicum in Bydgoszcz, Nicolaus Copernicus University in Toruń, 20 Świętojańska St., 85-077 Bydgoszcz, Poland; violetta.tomaszewicz@cm.umk.pl; 5 Student Scientific Club of Biochemistry and Bioorganic Chemistry, Department of Medical Biology and Biochemistry, Faculty of Medicine, Ludwik Rydygier Collegium Medicum in Bydgoszcz, Nicolaus Copernicus University in Toruń, 24 Karłowicza St., 85-092 Bydgoszcz, Poland; 316714@stud.umk.pl

**Keywords:** alcohol dependency, antioxidant therapy, dietary antioxidants, microbial interactions, oxidative stress, reactive oxygen species, skin disorders

## Abstract

Alcohol dependency is a complex and chronic condition that negatively impacts multiple organ systems, including the skin. A key pathological factor in this process is oxidative stress, leading to progressive cellular damage, chronic inflammation, and accelerated cutaneous aging. Alcohol metabolism generates reactive oxygen species (ROS), which overwhelm endogenous antioxidant defenses and contribute to a range of skin alterations, including nonspecific changes such as xerosis, erythema, and wrinkle formation, as well as inflammatory and neoplastic skin disorders. Additionally, alcohol-induced alterations of the skin microbiome may further exacerbate skin barrier dysfunction and inflammatory responses. This review explores the biochemical mechanisms and skin microbiome alterations linking alcohol-induced oxidative stress to skin damage and disease. Furthermore, it evaluates the therapeutic potential of antioxidant-based interventions, both natural and synthetic. Antioxidants may offer protective and regenerative effects by scavenging free radicals, modulating inflammatory responses, and enhancing skin barrier function. The paper aims to provide a comprehensive overview of the molecular and microbial interplay between alcohol, oxidative stress, and skin health, while identifying future directions for targeted antioxidant therapy in individuals with alcohol dependency.

## 1. Introduction

Alcohol use disorder (AUD) is a condition characterized by an impaired ability to stop or control alcohol consumption despite harmful social, occupational, or health-related consequences [1]. According to the International Classification of Diseases 11th Revision, ICD-11 (6C40.2), alcohol dependence involves a strong internal drive to drink, impaired control over alcohol use, and continued consumption despite negative consequences [2]. Alcohol-related disorders represent a major public health challenge. They are one of the leading causes of premature death and long-term disability, and their effects extend beyond the liver and nervous system to include the cardiovascular system, bones, the immune system, and the skin [3,4,5,6]. Chronic alcohol consumption, in particular, impairs immune defenses by disrupting lymphocyte function and damaging protective barriers, which increases vulnerability to bacterial and viral infections [3].

The skin is one of the organs susceptible to the effects of alcohol, serving as the first line of defense against environmental factors and pathogens. It comprises three main layers: the epidermis, dermis, and subcutaneous tissue. The outermost layer, the epidermis, consists of stratified epithelium that provides a primary protective function. The lipid matrix located in the stratum corneum plays a key role in maintaining the integrity of this barrier. This matrix is composed of ceramides, cholesterol, and free fatty acids, such as linoleic acid (linoleate), palmitic acid (palmitate), and oleic acid (oleate), which contribute to the skin’s structural stability, water retention, and defense against microbial penetration [7,8]. In the context of chronic alcohol abuse, weakening of the epidermal barrier and immune dysfunction lead to increased susceptibility to skin infections, particularly those of bacterial and viral origin. Infections caused by *Staphylococcus aureus* tend to present more severely in individuals with AUD, primarily due to alcohol-related immune deficits [9,10,11]. Alcohol use is associated with a higher incidence of inflammatory skin diseases. The most common dermatoses include rosacea, psoriasis, atopic dermatitis, and acne vulgaris [12,13,14,15,16,17]. In the latter, *Cutibacterium acnes* plays a key pathogenic role [18]. In patients with AUD, a higher risk of human papillomavirus (HPV) infection is also observed, which may contribute to the development of skin cancers [19,20]. A substantial portion of alcohol-related skin impairments is driven by oxidative stress and redox imbalance. In this context, bioactive dietary compounds with antioxidant properties are gaining increasing attention as a potential supportive strategy to mitigate alcohol-induced oxidative damage to the skin.

Oxidative stress is a natural component of metabolic processes occurring in the skin, such as cellular respiration and energy transformations [21,22]. In the skin, small amounts of reactive oxygen species (ROS) such as superoxide anion (O_2_^•−^), hydroxyl radical (^•^OH), hydrogen peroxide (H_2_O_2_), and molecular oxygen (O_2_) are continuously produced during normal physiological processes, including cellular respiration and energy metabolism [21,22]. Nitric oxide (^•^NO), classified as a reactive nitrogen species (RNS), is also generated in the skin and functions similarly to ROS by participating in redox signaling and the oxidative stress response [21]. The primary endogenous sources of ROS include mitochondria and NADPH oxidases (NOX) [23]. Mitochondria in skin cells, such as keratinocytes and fibroblasts, are significant oxygen consumers during ATP production. In these processes, oxygen undergoes electron transfer reactions that can generate ROS [22]. ROS can also originate from external sources. Ultraviolet (UV) radiation activates porphyrins, heme metabolites with photodynamic properties, leading to ROS formation in the skin [24]. Alcohol consumption has been associated with reduced levels of systemic antioxidants such as carotenoids, which may influence the skin’s oxidative balance and response to UV exposure [25]. Furthermore, inflammatory responses promote ROS production through the action of infiltrating leukocytes. These immune cells, once activated, produce reactive species including O_2_^•−^, nitric oxide (NO), and hypochlorite anion (ClO^−^) via enzymes such as myeloperoxidase and nitric oxide synthase (NOS) [21,22]. ROS levels can oxidize proteins, DNA, and lipids when they exceed the skin’s antioxidant capacity, leading to structural and functional cellular damage [26]. These molecular disturbances trigger the activation of signaling pathways including the mitogen-activated protein kinase (MAPK) cascade, nuclear factor kappa-light-chain-enhancer of activated B cells (NF-κB), and activator protein 1, which modulate inflammatory and stress responses [21]. Under physiological conditions, the skin is equipped with several antioxidant defense systems. These include enzymatic antioxidants such as catalase (CAT), glutathione reductase (GR), and superoxide dismutase (SOD) [22]. In addition, non-enzymatic antioxidants like vitamin C (ascorbic acid), vitamin E (α-tocopherol), ubiquinone (coenzyme Q10), and uric acid help neutralize excess ROS and maintain redox homeostasis in skin cells [21,22].

This review aims to explore the pathophysiological mechanisms associated with the effects of chronic alcohol consumption on the skin, with particular emphasis on oxidative stress as a central damaging factor. It also seeks to present the current state of knowledge regarding the use of both natural and synthetic antioxidant substances in the prevention and management of skin disorders observed in individuals with alcohol dependence. The work integrates biochemical, microbiological, dermatological, and nutritional perspectives to evaluate the potential of antioxidants as a complementary approach to conventional treatment strategies.

## 2. Alcohol-Induced Skin Damage Mechanisms: Oxidative Stress, Lipid Peroxidation, and Barrier Dysfunction

Alcohol consumption, both acute and chronic, leads to the increased production of ROS in various organs and tissues of the body, including the liver, brain, pancreas, gastrointestinal mucosa, and skin [27,28,29,30]. Alcohol dehydrogenase (ADH) and aldehyde dehydrogenase (ALDH) activity have been detected in human skin, suggesting that it may play a role in the extrahepatic metabolism of alcohol [27,31]. Nevertheless, most ingested ethanol is metabolized primarily in the liver and, to a lesser extent, in the stomach, through oxidative and non-oxidative pathways. In the oxidative pathway, ADH first converts ethanol into acetaldehyde, which is then further oxidized to acetate by mitochondrial ALDH2. Both enzymes utilize nicotinamide adenine dinucleotide (NAD^+^) as a cofactor, resulting in a shift in the redox potential [32]. During chronic alcohol consumption, increased ethanol concentrations induce the cytochrome P450 enzyme CYP2E1, which is present in both the endoplasmic reticulum and mitochondria, contributes to ethanol oxidation using reduced nicotinamide adenine dinucleotide phosphate (NADPH) as a cofactor, and generates superoxide anions, leading to the production of ROS and RNS [29]. Oxidative stress associated with ethanol metabolism results both from impaired antioxidant defenses and excessive production of ROS, particularly by the inducible enzyme CYP2E1, the mitochondrial electron transport chain, and activated phagocytes [33,34,35]. One of the key free radical products specific to ethanol metabolism is the hydroxyethyl radical (HER), which is generated via microsomal oxidation of ethanol involving CYP2E1. HER not only participates in lipid peroxidation, but has also been shown to induce mitochondrial permeability transition, leading to mitochondrial swelling, membrane potential collapse, and oxidation of membrane protein thiols, thereby impairing mitochondrial function and contributing to cell death via apoptosis and necrosis [34,36]. In addition, HER can alkylate proteins, forming a distinct group of antigens that do not cross-react with acetaldehyde-derived epitopes, thus contributing to the development of specific immunologic responses in alcoholic patients [37]. Localization of CYP2E1 to mitochondria enhances the local production of HER and ROS, particularly in hepatocytes, contributing to impaired mitochondrial integrity and function [34,35]. These reactive species do not remain confined to the liver; oxidative stress resulting from alcohol metabolism affects other tissues as well, including the skin. Keratinocytes, the predominant cell type in the epidermis, are rich in mitochondria and highly sensitive to redox imbalance [38]. Ethanol-induced production of HER and ROS may disrupt keratinocyte mitochondrial integrity, particularly under UV stress, thereby compromising skin barrier function and cellular viability. This oxidative stress damages lipids, proteins, and DNA, significantly reducing the efficiency of the skin’s antioxidant network and increasing susceptibility to UV-induced damage such as sunburn and skin cancer [33,38]. In the non-oxidative pathway, ethanol conjugates with various small molecules, such as fatty acids, leading to the formation of fatty acid ethyl esters (FAEEs) and phosphatidyl ethanol [32]. It is also important to note that alcohol exerts systemic effects beyond metabolism, particularly on the immune system. Disruption of the intestinal barrier and increased gut permeability can lead to bacterial and endotoxin translocation, triggering inflammation and increasing susceptibility to infections, further amplifying oxidative stress and tissue injury [39].

ROS can initiate the lipid peroxidation process, leading to cellular membrane damage. This process begins with the interaction between ROS and polyunsaturated fatty acids, resulting in the abstraction of a hydrogen atom and forming a lipid radical. Subsequent steps involve the addition of molecular oxygen to form a peroxyl radical, which reacts with another fatty acid, producing a lipid hydroperoxide and a new radical, thus propagating a chain reaction [40]. In alcoholics, excessive lipid peroxidation leads to the formation of toxic metabolites such as malondialdehyde (MDA) and 4-hydroxy-2-nonenal (HNE) [41]. Lipid peroxidation products react with cellular proteins, and the resulting modifications serve as sensitive biomarkers of alcohol-induced oxidative stress [42]. Structural and functional alterations of proteins can contribute to dysfunction of cellular membranes and intracellular structures [43]. Studies on human facial skin fibroblasts have shown that the amount of proteins modified by 4-HNE clearly increases with cellular age, indicating that 4-HNE may serve as a marker of oxidative damage accumulation in the skin [43]. Alterations in skin lipids caused by acute alcohol consumption lead to transepidermal water loss (TEWL), which is associated with intensified lipid peroxidation and damage to the structural lipids of the stratum corneum [27]. These changes persist for at least 2–4 weeks after alcohol cessation, suggesting lasting damage to the skin barrier [27]. When TEWL increases, self-repair mechanisms are triggered in the epidermis’s outermost layer (stratum corneum) and the granular layer. An initial inflammatory response leads to the release of cytokines such as tumor necrosis factor-alpha (TNF-α), interleukin-1 (IL-1), and interleukin-6 (IL-6), which stimulate keratinocyte proliferation and epidermal thickening. Concurrently, the production of skin lipids (e.g., ceramides, cholesterol) and components of the natural moisturizing factor, such as urea, lactic acid, and amino acids, increases, supporting stratum corneum restoration [44]. Alcohol intake reduces linoleate levels while increasing oleate and palmitate release into the bloodstream. This shift in fatty acid composition may lead to the formation of morphologically normal intercellular lamellae that are functionally impaired, contributing to an incomplete skin barrier [27]. Ethanol can penetrate the skin through the intercellular lipid layers of the stratum corneum, potentially disrupting the epidermal barrier and increasing its permeability to external substances [45,46]. The simultaneous action of alcohol and UV radiation enhances both the initiation and promotion of carcinogenesis processes [33]. In the presence of UV light, ethanol indirectly promotes the generation of ROS, partly through the photosensitizing effects of its metabolic products. The resulting ROS damage to DNA induces permanent structural changes and activates signaling pathways involved in inflammation and cell proliferation. Additionally, increased prostaglandin synthesis may intensify the inflammatory response, collectively creating favorable conditions for the development of skin cancer [21,33]. The skin, being particularly vulnerable to the effects of ROS, undergoes degradation of collagen and elastic fibers, leading to the loosening of both the dermis and subcutaneous tissue. ROS interfere with the synthesis of new collagen, which is responsible for rebuilding and strengthening skin structures as well as tissue regeneration. These changes result in slower wound healing [30].

## 3. Oxidative Stress and the Skin’s Biological Antioxidants in Redox Regulation

In keratinocytes, thiols such as glutathione (GSH) act as key antioxidants that regulate intracellular redox balance and protect cells from oxidative damage, especially in the stratum corneum [47]. Keratinocytes are the primary source of thiols in the epidermis due to their high production of GSH [48]. Thiols are among the first antioxidants to be depleted during oxidative stress, and their levels indicate the extent of cellular damage. GSH neutralizes reactive oxygen and nitrogen species, supports the activity of antioxidant enzymes like glutathione peroxidases (GPx) and glutathione S-transferases (GST), prevents irreversible protein oxidation through glutathionylation, and participates in detoxification through conjugation [47]. GSH exists in cells in a dynamic equilibrium between its reduced form, GSH, and its oxidized glutathione (GSSG), which reflects the cellular redox status. During the neutralization of ROS, GSH is oxidized to GSSG, which is subsequently reduced back to GSH by glutathione reductase (GR), ensuring the maintenance of redox homeostasis [49]. An essential component of this antioxidant system is GPx, an enzyme that, in cooperation with GSH, reduces organic hydroperoxides and H_2_O_2_ to non-toxic compounds, thereby limiting oxidative damage. This process occurs concurrently with the oxidation of GSH, making GPx a key factor in maintaining cellular redox homeostasis. CAT is another crucial antioxidant enzyme that decomposes H_2_O_2_, a product of reactions catalyzed by SOD, into water and oxygen. SOD, in turn, catalyzes the conversion of the superoxide anion (O_2_^•−^) into H_2_O_2_, which is subsequently detoxified. As a result, SOD plays a crucial role in protecting cells against oxidative stress, particularly under physiological condition [50].

Non-enzymatic compounds also play an essential role in protecting cells against oxidative stress. The group of compounds known as vitamin A includes retinol and its derivatives: retinal, retinoic acid (including the biologically active all-trans retinoic acid), retinyl esters, as well as specific carotenoids, such as β-carotene, which serve as provitamin A [51]. Vitamin A does not act as a classical antioxidant in a direct manner. Its protective function is mainly exerted through gene transcription regulation via all-trans retinoic acid, which activates antioxidant enzymes such as SOD, CAT, and GPx [52]. Both vitamin A and its provitamin, β-carotene, may play a significant role in shielding cells from oxidative stress, for example, by limiting lipid peroxidation [53]. Carotenoids, unlike vitamin A, function as direct antioxidants. Their action primarily involves the neutralization of singlet oxygen and peroxyl radicals. Singlet oxygen can be quenched in two ways: physically, by energy transfer without carotenoid degradation, or chemically, leading to permanent damage to the carotenoid molecule. Carotenoids can also interact with peroxyl radicals, forming stable resonance-stabilized radicals or oxidation products such as epoxides. These reactions may inhibit the spread of lipid peroxidation in cell membranes, though they do not always result in complete neutralization of oxidative stress. The antioxidant efficacy of carotenoids also depends on oxygen concentration; they act as effective antioxidants under low oxygen tension, but at higher levels, they may participate in pro-oxidative reactions [54]. Moreover, some oxidized carotenoid metabolites, such as ketocarotenoids, can activate the Nrf2 pathway and upregulate the expression of antioxidant enzymes, indicating an additional indirect protective role [55].

Vitamin C refers to L-ascorbic acid and its oxidized form, L-dehydroascorbic acid. Both forms possess antioxidant properties, and dehydroascorbate is actively converted back into ascorbate, preserving its biological functions [56]. In addition, this vitamin participates in the activation of various enzymes, enhances detoxification and immune defense, and counteracts lipid peroxidation processes [51]. Its antioxidant action involves modulating the activity of NADPH oxidase and NOS, thereby protecting the vascular endothelium from oxidative damage [56]. Vitamin C also contributes to the regeneration of hydrophobic antioxidants, such as α-tocopherol and β-carotene, from their radical forms [51,57]. Moreover, GSH plays a key role in regenerating vitamin C, enhancing its antioxidant efficiency [56]. Vitamin C deficiency, especially in individuals with chronic alcohol abuse, can impair connective tissue synthesis, leading to poor skin condition, delayed wound healing, and increased fragility of blood vessels [51].

Vitamin D includes two primary forms: cholecalciferol (vitamin D_3_), found primarily in animal-based foods, and ergocalciferol (vitamin D_2_), present in plant-based products, mushrooms, and yeast. Its biologically active form in the body is 1,25-dihydroxycholecalciferol (calcitriol), while 25-hydroxycholecalciferol (25(OH)D) serves as the primary indicator of vitamin D levels in the blood [51]. Vitamin D plays a protective role in the skin by acting as an antioxidant. It is synthesized locally in keratinocytes and converted into its active form. It binds to the vitamin D receptor and regulates the expression of genes involved in oxidative stress response and the maintenance of the epidermal barrier [58].

Vitamin E (α-tocopherol) is a group of fat-soluble compounds with antioxidant properties, comprising eight forms: tocopherols and tocotrienols (including α-, β-, γ-, and δ-isomers), which differ in their saturation and the distribution of methyl groups along the side chain [51]. Among them, α-tocopherol is considered the most biologically active form in humans and is most commonly found in dietary supplements [59]. The primary antioxidant function of vitamin E is to protect membrane lipids, especially polyunsaturated fatty acids, from peroxidation. This vitamin safeguards cellular structures, including the membranes of mitochondria, the endoplasmic reticulum, and the plasma membrane, against the damaging effects of ROS [51,59,60]. It is a key component of the antioxidant network, including vitamin C, GSH, and NADPH, working together to maintain redox balance in the body [60]. Vitamin E is primarily an antioxidant in hydrophobic environments, neutralizing free radicals generated during metabolic processes [51].

Riboflavin (vitamin B2) is a water-soluble vitamin that exists in three forms: as free riboflavin and as biologically active coenzymes, flavin adenine dinucleotide and flavin mononucleotide, which function as essential cofactors for numerous redox enzymes [51,61,62]. This vitamin plays a crucial, though often overlooked, role in protecting cells from oxidative damage by supporting antioxidant defense mechanisms dependent on these flavin-based coenzymes. One of its key actions in the context of oxidative stress is to help the activity of GR [62]. Riboflavin deficiency increases the level of lipid peroxidation products such as MDA, which indicates increased oxidative damage to cell membranes. Numerous animal studies have demonstrated that insufficient riboflavin intake is associated with decreased activity of antioxidant enzymes, including SOD, CAT, and GPx, which leads to a disruption of redox balance and the accumulation of ROS [61,62]. It is also suggested that riboflavin may act as an antioxidant independently of the glutathione cycle, either by directly scavenging free radicals or by enhancing the function of other antioxidants such as vitamin C [62].

Vitamin B3 (niacinamide), in the form of nicotinamide, is a component of key coenzymes such as NAD^+^, its reduced form (NADH), nicotinamide adenine dinucleotide phosphate (NADP^+^), and NADPH. These coenzymes play a crucial role in metabolic pathways and redox reactions within cells. NAD^+^ acts as an electron acceptor in glycolysis, the citric acid cycle, and β-oxidation of fatty acids, while NADH donates electrons in the mitochondrial respiratory chain. NADP^+^ is an electron acceptor in the pentose phosphate pathway and other anabolic processes. In contrast, NADPH is essential for glutathione regeneration and the activity of enzymes such as cytochrome P450, NOS, and GR [63].

Zinc (Zn) is a cofactor for numerous enzymes, particularly those classified as metalloproteins, including hydrolases, transferases, oxidoreductases, ligases, and isomerases. It is estimated that up to 10% of all proteins in the human body contain zinc as an integral component. Notably, many of these proteins serve regulatory functions, influencing key biological processes [51]. Zinc also plays a vital role in maintaining the redox balance of cells. Under conditions of zinc deficiency, a significant decrease in GSH levels is observed, suggesting that zinc supports the maintenance of intracellular reducing potential [64]. Zinc supplementation in human skin fibroblasts results in a substantial increase in its intracellular concentration, as well as a reduction in the levels of lipid peroxidation products, such as MDA, confirming its protective properties against oxidative damage [65]. Depending on its concentration, zinc acts as both an antioxidant and a pro-oxidant [65]. It regulates GSH levels in skin keratinocytes; its deficiency leads to decreased GSH content and an increased efflux of GSH from cells, weakening intracellular antioxidant defense. Excess zinc may render cells more susceptible to damage induced by H_2_O_2_ or other potent oxidizing agents. Moreover, zinc stabilizes the structure of enzymes such as Cu/Zn-superoxide dismutase, protecting thiol groups from oxidation [64]. Additionally, zinc inhibits the activity of the enzyme NADPH oxidase, thereby limiting the formation of free radicals, and induces the synthesis of metallothioneins, which neutralize reactive oxygen species and protect cells from oxidative stress. Zinc also influences the expression of glutamate cysteine ligase, a key enzyme for glutathione synthesis, through activation of the Nrf2 signaling pathway, further enhancing the antioxidant potential of cells. Under oxidative stress conditions, zinc is released from its complexes with metallothionein and redirected to sites requiring protection, further strengthening its antioxidant action [66].

Selenium (Se) is an essential trace element whose biological role arises primarily from its presence in the structure of selenoproteins [51]. A characteristic feature of selenoproteins is the presence of selenocysteine, which is incorporated into the active sites of enzymes that play a key role in maintaining cellular redox balance. The essential selenoenzymes include GPx and thioredoxin reductase (TrxR), which neutralize peroxides and regenerate proteins oxidized under oxidative stress. Selenoproteins protect DNA, lipids, and proteins from damage through these mechanisms and regulate metabolic processes, immune function, and hormonal balance [23].

Coenzyme Q_10_ (CoQ_10_) is an endogenous lipophilic quinone, ubiquitous in biological membranes, with antioxidant and bioenergetic properties [67]. It plays a dual role as a key component of the electron transport chain in cellular respiration and a potent antioxidant [67,68]. Its reduced form acts as a major membrane-bound antioxidant, protecting lipids, DNA, and proteins from oxidative stress. CoQ_10_ also helps limit the production of mitochondrial reactive oxygen species and supports uncoupling protein function [68]. Its concentration tends to decline with age in various tissues, including the skin [67].

Polyphenolic compounds, such as the flavonoids quercetin and rutin, and the stilbene resveratrol, have gained significant interest in pharmaceutical research due to their broad spectrum of beneficial biological properties. Quercetin plays an important role in skin protection, acting as a potent antioxidant that reduces the levels of ROS, lipid peroxides, and MDA, while increasing GSH levels and the activity of antioxidant enzymes such as SOD and CAT. In addition, it exhibits anti-inflammatory, anti-aging, wound-healing, skin-brightening, and anticancer effects, making it a promising therapeutic agent in dermatology [69]. Resveratrol also shows strong antioxidant and anti-inflammatory properties, and further demonstrates neuroprotective, antidiabetic, and cardioprotective effects. Its ability to scavenge free radicals and chelate metal ions is attributed to the presence of hydroxyl groups. Moreover, it supports intracellular antioxidant defense by reducing ROS production, limiting lipid peroxidation, and enhancing the activity of GSH, SOD, and CAT [70]. Rutin (also known as rutoside), also exhibits strong antioxidant and cytoprotective properties, particularly in the context of UV-induced skin damage. It enhances the expression of antioxidant enzymes such as SOD and thioredoxin reductase, and supports redox balance by activating the Nrf2 signaling pathway [71,72]. Table 1 summarizes selected antioxidants that contribute to skin health and redox regulation in individuals affected by alcohol-induced oxidative stress.

## 4. The Impact of Alcohol Consumption on Oxidative Stress and Microbial Factors in Skin Diseases

Atopic dermatitis is a chronic inflammatory skin disease characterized by epidermal barrier dysfunction and a predominance of the T helper type 2 (Th2-type) immune response [93]. Alcohol consumption during pregnancy increases the risk of developing atopic dermatitis in offspring, most likely due to an enhanced skewing of the immune response towards the Th2 profile and elevated levels of immunoglobulin E [12]. In adults, excessive alcohol intake may be associated with more severe forms of atopic dermatitis; however, moderate alcohol consumption does not appear to have an apparent effect on the course of the disease [12,13]. In atopic dermatitis, increased oxidative stress is observed, as indicated by elevated levels of ROS and lipid peroxidation products such as MDA and 4-hydroxynonenal (4-HNE), particularly in the skin and, to a lesser extent, in body fluid [93,94]. Mitochondrial dysfunction in keratinocytes may lead to excessive production of H_2_O_2_ and the release of cytochrome c, potentially intensifying the inflammatory response and contributing to structural damage of the epidermis. Moreover, oxidative stress is associated with DNA damage, as reflected by elevated levels of 8-hydroxy-2′-deoxyguanosine in both lesional and clinically unaffected skin of patients with atopic dermatitis [94]. Both lesional and clinically unaffected skin in patients with atopic dermatitis show increased colonization by the Gram-positive bacterium *S. aureus*. This may result from preferential expression of bacterial receptors in atopic skin or impairments in host defense mechanisms [93]. In individuals with alcohol use disorder, skin infections tend to be more severe and more frequently result in complicated skin and soft tissue infections. Interestingly, this is not necessarily due to higher rates of *S. aureus* colonization, but may reflect alcohol-related impairments in immune defense, which exacerbate the impact of colonizing pathogens [9]. Colonization of the skin by *S. aureus* further exacerbates oxidative stress by activating monocytes and increasing ROS production, leading to additional damage of the epidermal barrier and intensifying the inflammatory state [93].

Colonization of the skin by *S. aureus*, accompanied by increased oxidative stress and inflammatory response, plays a significant role in skin damage and regeneration. Chronic alcohol abuse is a predisposing factor for necrotizing wound infections and delayed healing of damaged skin. Moreover, it increases the risk of oxidative skin damage and sunburn, particularly following UV exposure [27,33]. The wound healing process proceeds through four phases: hemostasis (cessation of bleeding), inflammation, proliferation (cellular rebuilding), and remodeling (organization of new tissue). Immediately after injury, platelets activate clot formation, and the body initiates an inflammatory response involving neutrophils and macrophages. In the following days, epidermal cells proliferate, new blood vessels form, and fibroblasts produce extracellular matrix. The final phase includes tissue reorganization and wound closure [44]. Alcohol impairs the function of dermal fibroblasts, which are responsible for tissue regeneration and the secretion of growth factors essential for the repair process. Exposure of skin cells to ethanol decreases the strength of healing wounds. It impairs immune cell activity [39]. Chronic ethanol exposure reduces both the number and functional capacity of dendritic epidermal T cells (DETCs) and dermal γδ T cells (γδT17 cells), which are critical for maintaining cutaneous immune defense and initiating responses to tissue injury. Ethanol impairs their ability to produce pro-inflammatory cytokines such as TNFα, and reduces the expression of activation and adhesion molecules necessary for effective immune cell recruitment and signaling. Additionally, chronic alcohol consumption alters the expression of chemokine receptors, impairs epidermal immune surveillance, and weakens antibacterial responses, which may contribute to increased susceptibility to skin infections caused by *S. aureus* [10,11].

Thiol/disulfide homeostasis plays a key role in regulating the skin’s redox balance and antioxidant defense. In patients with rosacea, this balance shifts significantly toward disulfides, which reflects increased oxidative stress and may contribute to the disease’s inflammatory pathogenesis [95]. In individuals with rosacea, reduced serum activity of antioxidant enzymes such as paraoxonase and arylesterase suggests impaired systemic antioxidant defense mechanisms. This enzymatic deficiency is accompanied by elevated levels of lipid hydroperoxides, a marker of lipid peroxidation, further indicating intensified oxidative stress [96]. Additionally, lower activities of enzymes like SOD and CAT have been observed further supporting the presence of oxidative imbalance, particularly in more active stages of the disease [97]. Epidemiological studies conducted in the United States and the United Kingdom have shown that alcohol consumption is associated with a modestly increased risk of developing rosacea [14,15]. Patients with rosacea have been reported to frequently present with gastrointestinal conditions such as small intestinal bacterial overgrowth, which may be more prevalent among individuals who consume alcohol [98,99].

Alcohol use disorders occur significantly more frequently in patients with psoriasis than in individuals with non-inflammatory skin diseases, and in those with moderate psoriasis, a positive correlation has been observed between the amount of alcohol consumed and the severity of skin symptoms, particularly among women [16,17]. Although the mechanisms underlying the development of psoriasis are not fully understood, oxidative stress and ROS are believed to play a significant role in its pathogenesis [12]. In the pathogenesis of psoriasis and psoriatic arthritis, elevated levels of oxidative stress biomarkers have been identified, including MDA, advanced glycation end-products, and advanced oxidation protein products, along with decreased activity of antioxidant enzymes such as SOD, CAT, and GPx [50]. Excessive ROS simultaneously activate NF-κB and MAPK signaling pathways, leading to increased production of pro-inflammatory cytokines such as TNF-α, interleukin-1 beta (IL-1β), interleukin-6 (IL-6), and IL-17. Alcohol consumption is a known cause of increased intestinal permeability, facilitating the translocation of gut microbiota into the bloodstream [100]. Most microorganisms inhabiting the gut are Gram-negative bacteria, whose cell wall contains lipopolysaccharide (LPS), a compound with strong pro-inflammatory properties. The entry of LPS into the bloodstream can lead to immune system activation and the development of systemic inflammation [101]. Elevated serum levels of pro-inflammatory cytokines (such as TNF-α, IL-1β, IL-6, and interleukin-8) and endotoxins like LPS have been implicated in exacerbating psoriatic inflammation. Both LPS and these cytokines can disrupt epidermal barrier function and stimulate keratinocyte proliferation and inflammatory signaling characteristic of psoriasis [45].

Acne vulgaris is a chronic inflammatory disease of the pilosebaceous follicles with a multifactorial pathogenesis. Key contributing factors include hyperplasia of sebaceous glands and increased sebum production, abnormal keratinization of the pilosebaceous duct, colonization by *C. acnes* (a Gram-positive facultative anaerobic bacterium, formerly known as *Propionibacterium acnes*), and the development of an inflammatory response. These mechanisms interact and play a crucial role in the formation of acne lesions in both adolescents and adults [18,102]. Alcohol consumption may be a risk factor for the development of acne, with its impact potentially related to alterations in the skin microbiome, increased oxidative stress, and the intensification of inflammatory processes within the pilosebaceous unit, promoting the appearance of mild acne lesions [12]. The severity of acne vulgaris positively correlates with increased oxidative stress, as evidenced by elevated levels of MDA and reduced activity of SOD, suggesting the exhaustion of antioxidant defense mechanisms in severe cases [103]. *C. acnes* also plays a significant role in enhancing oxidative stress at the site of inflammation, as neutrophils activated by the bacterium generate ROS, leading to cellular structure damage [102]. It should be noted that *C. acnes* secretes the antioxidant protein RoxP, which neutralizes ROS and protects skin cells from oxidative stress [104]. In the pathogenesis of acne vulgaris, especially in adult forms, mites also play an essential role [105]. Alcohol consumption is significantly associated with increased *Demodex* spp. positivity in patients with post-adolescent acne, but not in those with adolescent acne [105].

Melanoma is a malignant tumor originating from melanocytes, most commonly found in the skin, but it can also develop in mucous membranes and internal organs. Melanomas may develop not only in chronically sun-exposed skin but also in acral locations (e.g., palms, soles), mucosal sites, and within congenital or blue nevi [106,107]. Its incidence is increasing and is associated with genetic and phenotypic predispositions and environmental factors. Epidemiological studies suggest that alcohol consumption may be a risk factor for melanoma development. An analysis conducted within the European Prospective Investigation into Cancer and Nutrition study showed that both current and lifetime alcohol consumption were positively correlated with melanoma risk in men, particularly with high consumption of spirits and white wine [108]. Oxidative stress and disturbances in redox homeostasis play a key role in the development and progression of this malignant tumor. Excessive ROS levels in melanocytes, resulting from melanin synthesis and UVA radiation exposure, lead to DNA damage and the formation of tumor-initiating cells [109]. Melanoma cells adapt to high levels of ROS by increasing the activity of antioxidant systems such as SOD, CAT, and GPx, which promotes their survival, development of treatment resistance, and ability to form metastases [110].

Approximately 98% of all skin cancers are basal cell carcinoma (BCC) and squamous cell carcinoma (SCC), which are the most common malignant tumors occurring in individuals with fair skin [111]. The development of BCC and SCC is primarily associated with chronic exposure to UV. However, other environmental factors, such as infection with human papillomavirus (HPV) in the case of SCC and genetic predispositions, also play an essential role [12,111,112]. HPVs are small, non-enveloped DNA viruses that show tropism for epithelial cells. They mainly infect basal and squamous cells, and their replication and translation occur as the infected cells differentiate [112]. Notably, alcohol consumption may further increase the risk of HPV infection. Studies have shown that high alcohol intake is associated with a higher prevalence of HPV infections, independently of sexual behaviors or smoking [19]. Oxidative stress, potentially enhanced by alcohol metabolism, may promote the integration and persistence of the HPV genome, contributing to tumor progression. However, the specific dose-dependent relationship between alcohol consumption and risk of BCC/SCC remains to be fully established [20]. ROS damage DNA, lipids, and proteins, promoting mutations and initiating carcinogenic processes [113]. Patients with BCC and SCC exhibit elevated levels of oxidative stress markers, such as lipid peroxidation products and oxidized proteins, as well as increased DNA damage. At the same time, they show reduced activity of antioxidant enzymes, including SOD, CAT, and GPx, indicating impaired defense mechanisms against oxidative stress [114]. Oxidative stress also activates the MAPK and NF-κB signaling pathways, supporting cell proliferation, angiogenesis, and tumor resistance to apoptosis [115].

## 5. Evidence from In Vitro and Animal Studies

In vitro studies and experiments using animal models have provided valuable insights into the pathogenic mechanisms underlying alcohol-induced skin damage. Recent evidence links alcohol consumption to increased susceptibility to sunburn and skin cancer. A pilot study in human volunteers demonstrated that alcohol intake significantly reduced skin carotenoid levels and the minimal erythema dose (MED), indicating impaired antioxidant capacity—an effect not observed when alcohol was consumed with orange juice. Supporting these findings, in vitro studies using human adult low-calcium high-temperature keratinocytes have shown that ethanol exposure increases ROS, activates NF-κB signaling, and upregulates pro-inflammatory cytokines such as IL-1β and TNF-α. These changes intensify inflammation and compromise the epidermal barrier [33]. Concurrently, reduced expression of key structural skin proteins, including filaggrin and loricrin, has been observed, potentially disturbing epidermal water–lipid homeostasis and increasing permeability to environmental irritants [33]. Ethanol also significantly stimulates lipogenesis in human sebocytes, particularly via non-oxidative metabolic pathways that generate FAEEs. In contrast, oxidative ethanol metabolism does not appear to contribute to this effect. Furthermore, ethanol impairs mitochondrial ATP production by reducing oxygen consumption, while glycolytic ATP generation remains unaffected, indicating that ethanol-induced lipogenesis is oxygen-independent. These metabolic alterations provide a mechanistic link between chronic alcohol consumption and the development or exacerbation of acne, suggesting new therapeutic targets for acne vulgaris in individuals with alcohol use disorder [116]. Acute ethanol exposure negatively impacts fibroblast function by reducing proliferative capacity and downregulating genes involved in extracellular matrix production, including collagen I, collagen III, and lysyl oxidase. Both in vitro and in vivo studies have shown that ethanol weakens wound strength, likely through diminished collagen synthesis and lysyl oxidase activity, offering a mechanistic explanation for the increased incidence of wound complications in alcohol-exposed individuals [117]. Long-term ethanol administration (12–16 weeks) in mice leads to numerical and functional impairments in several skin-resident T cell subsets, including DETCs and γδT17 cells. These cells show reduced density, proliferation, and cytokine production, indicating compromised skin immune surveillance and helping to explain the increased susceptibility to skin infections observed in individuals with chronic alcohol abuse [11]. Similarly, chronic ethanol consumption in C57BL/6 mice (5% ethanol for 10 weeks) exacerbated psoriasiform dermatitis induced by topical application of imiquimod, a Toll-like receptor 7 agonist commonly used to model psoriasis in mice. Histological and transcriptomic analyses revealed that alcohol promotes subclinical skin inflammation—likely via Th17-mediated pathways—predisposing to amplified inflammatory responses. These findings underscore the importance of managing alcohol intake in patients with psoriasis [118]. Another investigation evaluated the long-term effects of ethanol on oxidative stress in aging rats. Male rats aged 15, 20, and 24 months received intraperitoneal ethanol (1.5 g/kg/day) or saline for 13 weeks, followed by a 2-month withdrawal period. While age-related declines in antioxidant enzyme activity (SOD, CAT, GPx) were observed in controls, ethanol-treated rats showed no such decline, suggesting a persistent, age-interactive oxidative effect of low-dose ethanol exposure [119]. Ethanol also impairs the migratory capacity of cutaneous dendritic cells (CDCs) without altering their baseline numbers in the dermis. In mice receiving ethanol in drinking water for over 16 weeks, fluorescein isothiocyanate sensitization and flow cytometry revealed significantly reduced CDC migration to draining lymph nodes. Mechanistically, this was linked to inadequate downregulation of chemokine receptors and adhesion molecules and insufficient upregulation of matrix metalloproteinases—all essential for effective migration. Skin explant cultures further indicated altered local cytokine production in response to ethanol. The partial restoration of CDC migration following TNF-α supplementation pointed to intrinsic and microenvironmental deficits. These findings provide mechanistic insight into compromised skin immune surveillance in chronic alcohol exposure [120]. Finally, alcohol has been shown to impair angiogenesis, as evidenced by decreased vascular endothelial growth factor expression and reduced microvascular density in the skin, ultimately slowing wound healing and tissue regeneration [121].

A range of in vitro and animal model studies has elucidated the complex mechanisms by which ethanol impacts skin physiology. Ethanol exposure increases oxidative stress, disrupts skin barrier integrity, and promotes keratinocyte pro-inflammatory signaling. It stimulates sebocyte lipogenesis through oxygen-independent pathways, impairs fibroblast proliferation and collagen synthesis, and weakens wound healing. Long-term alcohol intake alters skin immunity by suppressing T cell subsets and dendritic cell function, contributing to increased susceptibility to infections and inflammatory skin conditions. Additionally, chronic ethanol exposure has age-interactive effects on antioxidant enzyme activity and reduces angiogenesis, further compromising tissue repair. These findings provide mechanistic insight into the detrimental effects of alcohol and support its role as a modifiable factor in skin pathology.

## 6. Bioactive Food Components with Antioxidant Properties in Skin Protection Among Individuals with Alcohol Use Disorders

In the context of alcoholism, several additional factors contribute to deteriorating skin health beyond oxidative stress alone. Malnutrition and associated micronutrient deficiencies—including zinc, selenium, and vitamins C, E, B_2_, and B_3_—are common in individuals with alcohol use disorder and are known to impair keratinocyte proliferation, skin barrier integrity, and the resolution of inflammation. Liver dysfunction further exacerbates these effects by impairing the hepatic metabolism of key vitamins, hormones, and detoxification enzymes essential for cutaneous homeostasis and immune function [122,123]. Other relevant contributors include intestinal malabsorption, chronic dehydration, and endocrine imbalance (e.g., altered cortisol and sex hormone levels), which collectively aggravate tissue-level oxidative stress and impede proper epidermal regeneration [124,125]. These overlapping metabolic and systemic disturbances significantly impact the clinical outcomes of skin-directed therapies and must be accounted for when assessing treatment efficacy.

A wide variety of natural foods contain bioactive compounds with strong antioxidant potential. They are increasingly recognized for their role in supporting skin health, particularly under conditions of oxidative stress such as chronic alcohol exposure. These compounds include essential vitamins (C, E, A, D, B2, and B3), carotenoids (notably β-carotene), polyphenols (such as resveratrol, quercetin, and rutin), and trace elements like selenium and zinc. Each of these nutrients contributes to the neutralization of ROS, helps maintain the structural integrity of the skin barrier, and modulates inflammatory processes, which are often dysregulated in individuals with alcohol use disorders [21,74,126,127].

Vitamin C is abundant in fresh fruits and vegetables and is essential to maintaining skin structure and resilience. Primary dietary sources include citrus fruits (such as oranges, grapefruits, and lemons), bell peppers, blackcurrants, strawberries, parsley, broccoli, and Brussels sprouts. These foods are essential in the diets of individuals with alcohol use disorders, who frequently have a reduced intake of fresh produce. Vitamin C supports the biosynthesis of collagen, which is crucial for maintaining skin firmness, and contributes to the regeneration of oxidized vitamin E, thereby reinforcing antioxidant defense within the lipid phase of cell membranes [73,74]. Chronic alcohol use is frequently associated with vitamin C deficiency, primarily due to inadequate dietary intake. This deficiency contributes to impaired skin barrier function, delayed wound healing, and increased vascular fragility. Restoring adequate intake through vitamin C-rich foods can support tissue regeneration and improve microvascular integrity [73,128].

Vitamin E, a fat-soluble antioxidant, is obtained almost exclusively from plant-based, lipid-rich foods. Primary dietary sources include unrefined vegetable oils (such as sunflower, safflower, wheat germ, and olive oil), nuts (almonds, hazelnuts), seeds (particularly sunflower seeds), and leafy green vegetables (including spinach and kale). Vitamin E protects polyunsaturated fatty acids within skin cell membranes from oxidative damage. Individuals with AUD often consume insufficient amounts of these foods, leading to vitamin E deficiency and increased lipid peroxidation. Incorporating whole-food sources of vitamin E into the diet can help reduce skin inflammation, support cellular membrane integrity, and slow the progression of photoaging. Moreover, the synergistic intake of foods rich in vitamins C and E enhances antioxidant protection across the skin’s hydrophilic and lipophilic compartments [77,78,79,129].

Carotenoids, including β-carotene, lycopene, lutein, and zeaxanthin, are naturally occurring pigments in colorful fruits and vegetables. Primary dietary sources include carrots, pumpkins, sweet potatoes, apricots, mangoes, and cantaloupe. These compounds function both as precursors to vitamin A and as potent quenchers of singlet oxygen, thereby contributing to antioxidant defense in the skin. In individuals with chronic alcohol use, carotenoid levels are often reduced due to insufficient dietary intake and increased oxidative degradation. This depletion impairs their natural photoprotective capacity and contributes to premature skin aging. Regular consumption of carotenoid-rich foods contributes to maintaining healthy skin tone, supporting collagen integrity, and enhancing the skin’s overall antioxidant defense capacity [90,91,92]. Polyphenols, a diverse group of potent dietary antioxidants, are particularly effective when consumed as part of whole foods. Compounds such as resveratrol (found in red grapes, wine, blueberries, and peanuts) and quercetin (present in onions, apples, broccoli, and tea) exhibit strong anti-inflammatory effects and act as scavengers of ROS. These polyphenols modulate key inflammatory signaling pathways, including NF-κB and MAPK, promoting skin cell regeneration. Diets rich in polyphenols have been shown to reduce erythema, enhance skin hydration, and improve epidermal barrier integrity, particularly in the context of alcohol-induced skin damage [85,86].

Zinc is a critical trace element for keratinocyte proliferation, wound healing, enzymatic antioxidant defense, and immune modulation. It is naturally present in various whole foods, including seafood (particularly oysters, shrimp, and crab), lean meats, eggs, dairy products, whole grains, legumes, seeds (especially pumpkin and sesame), and nuts. Zinc deficiency is frequently observed in individuals with AUD, primarily due to reduced dietary intake, alcohol-induced malabsorption, and increased urinary excretion. This deficiency is associated with impaired epidermal regeneration, delayed wound healing, heightened susceptibility to infections, and the development of eczematous or dermatitis-like skin lesions. Ensuring adequate intake of zinc-rich whole foods plays a fundamental role in maintaining skin integrity, enhancing barrier function, and reducing inflammation in this high-risk population [130,131,132].

Selenium is a trace element essential for the activity of selenoproteins such as glutathione peroxidase, which help protect skin lipids from oxidative stress. Key dietary sources include fish (tuna, sardines, salmon), shellfish, eggs, whole grains, and Brazil nuts. Selenium deficiency is common among individuals with alcohol use disorders due to liver dysfunction and malnutrition. It impairs keratinocyte proliferation, weakens immune responses, and promotes oxidative damage in the skin. Adequate selenium intake through whole foods supports skin regeneration and barrier function [87,88].

GSH, although synthesized endogenously, depends heavily on dietary precursors to maintain optimal intracellular levels. Cruciferous vegetables such as broccoli, cabbage, Brussels sprouts, cauliflower, and kale are particularly rich in glucosinolates, which release bioavailable sulfur compounds essential for GSH biosynthesis. Additional dietary sources that support GSH production include garlic, onions, leeks, shallots, avocados, spinach, asparagus, and legumes. In individuals with alcohol use disorders, chronic ethanol consumption leads to elevated oxidative stress and impaired hepatic function, both of which contribute to the depletion of intracellular GSH stores. This reduction compromises antioxidant defenses, accelerates skin aging, and promotes pro-inflammatory skin processes. Regular consumption of sulfur-containing vegetables, alongside vitamin C-rich foods that help regenerate oxidized GSH, may support glutathione homeostasis and enhance the skin’s detoxification capacity [80,81].

Integrating antioxidant-rich, whole-food sources into the diet of individuals with alcohol use disorders is a practical and evidence-based strategy for improving skin health. Bioactive compounds such as vitamins C and E, carotenoids, polyphenols, trace elements like zinc and selenium, and glutathione precursors are complementary in neutralizing oxidative stress, supporting dermal barrier integrity, reducing inflammation, and promoting skin regeneration. Given the high prevalence of nutritional deficiencies and oxidative imbalance in this population, dietary interventions focused on antioxidant support should be considered a fundamental component of dermatologic and systemic care. Future guidelines should emphasize food-based approaches as an adjunct to clinical therapies in alcohol-related skin disorders.

To ensure a balanced perspective, this review includes both natural and synthetic antioxidants. Natural antioxidants such as vitamins C and E, carotenoids, and polyphenols are primarily found in food sources and plant-derived topical preparations. In contrast, synthetic antioxidants—including ethylated ascorbic acid and stabilized forms of coenzyme Q10—are designed to enhance skin penetration and pharmaceutical stability. Although differences exist in pharmacokinetics and clinical application, both groups show potential in reducing oxidative stress and supporting the epidermal barrier in alcohol-related dermatoses. Comparative studies suggest that natural compounds offer multitargeted benefits and better tolerability, whereas synthetic agents often exhibit superior dermal penetration and shelf stability [133,134,135]. Further head-to-head clinical trials are needed to evaluate their relative efficacy in this specific context.

## 7. Systemic Antioxidant Supplementation in Alcohol Use Disorder: Clinical and Nutritional Considerations

The animal models showed a chance of alcohol dependence therapy consolidation by the integrative supplementation of antioxidants. The taking of resveratrol through metabolite formation can play a protective role by inhibiting ROS and modulating the brain-derived neurotrophic factor involved in hepatic disruption induced by chronic alcohol consumption. The protective effect could reinforce the potential use of resveratrol as a dietary supplement to prevent damage associated with long-lasting alcohol abuse [136]. Previously, clinical studies showed the positive effects after supplementation with antioxidants (e.g., vitamins and minerals). The longitudinal studies show that the serum levels of vitamin C and carotenoids significantly increased. The retinol and α-tocopherol concentrations decreased, and selenium and zinc were at the same level. The serum indicators were improved in the supplements compared to the placebo group for α-tocopherol, vitamin C, β-carotene, selenium, and zinc [137].

The supplementation of antioxidants helps improve the health status of hospitalized patients with alcohol dependence. The supplementation may support the therapy of the withdrawal syndrome. In this stage of treatment, it is essential to use nutrition interventions like nutrient supplements to ameliorate inadequacies. Nutrition interventions may include supplementation with thiamine, multivitamins, amino acids, probiotics, magnesium, or educational interventions [138]. The following elements are antioxidant compounds too [138]. Subsequent studies showed that antioxidants play a role in the neonatal neuroprotection if the pregnant mother has alcoholism. The trans-resveratrol supplementation could not reverse the deleterious effects of hypoxia–ischemia coupled with maternal alcohol dependence. The trans-piceatannol supplementation led to a recovery of all sensorimotor and cognitive functions. The authors of these studies suggested that this neuroprotection was obtained with a dose of trans-piceatannol corresponding to the consumption of a single passion fruit per day for a pregnant woman [139].

The above data highlight the importance of antioxidants in the therapy of alcohol dependence. Our clinical experience confirms the presence of dermatoses in the addicted patients, shown especially by symptoms like itchy skin, dry skin, changes in skin color (e.g., spots).

One of the studies showed clinical problems like “a latent scurvy”. The latent scurvy include such as weakness, leg pain, and muscle aching as well as skin purpura, petechiae, or hyperkeratosis of the legs (it is connected with collagen metabolism disturbance—the cartinine, requiring vitamin C for its hydroxylation, is an essential cofactor in the transport of long-chain fatty acid into mitochondrial matrix). The authors of the above studies suggested the vitamin C supplementation and dietary recommendation of eating fresh vegetables and fruit in patients with alcohol dependence that suffer from a latent scurvy [140].

At the moment and in the future, alcohol dependence therapy consolidation by the integrative supplementation of antioxidants plays an essential role in supporting and protecting against the side effects of heavy alcohol drinking. It is necessary in the context of the diagnosis of deficits and disturbances of alcohol drinking, one of the first symptoms of which is dermatoses [141].

## 8. Therapeutic Potential and Future Directions

Given the central role of oxidative stress in alcohol-induced skin damage, antioxidant-based formulations represent a promising frontier in both dermatocosmetic and pharmaceutical interventions. Numerous active compounds with redox-regulating properties are now incorporated into topical preparations designed to restore skin barrier function, reduce inflammation, and improve dermal regeneration in alcohol-affected individuals. Topical application of ascorbic acid in concentrations ranging from 10 to 20% has been shown to enhance collagen synthesis, reduce hyperpigmentation, and neutralize ROS, making it particularly useful in counteracting the oxidative damage associated with chronic alcohol consumption [33]. Commercial products such as SkinCeuticals C E Ferulic^®^ (New York, NY, USA) and La Roche-Posay Pure Vitamin C10 Serum have demonstrated efficacy in improving skin texture and reducing signs of aging caused by environmental and metabolic stressors, including ethanol metabolism. Vitamin E is another key lipid-soluble antioxidant used in dermatology for stabilizing cellular membranes and mitigating lipid peroxidation. Combined formulations, such as Obagi Professional-C Serum 20% (vitamin C + E), enhance antioxidant synergy and photoprotection [59,60]. CoQ_10_ has gained recognition in anti-aging dermatology due to its ability to support mitochondrial function and decrease the appearance of wrinkles and oxidative dermal fatigue. Products like Eucerin Q10 Active Day Cream are often recommended for skin under chronic oxidative burden, including that related to alcohol abuse [142]. Niacinamide, with anti-inflammatory and sebum-regulating properties, has been proven effective in reducing erythema, improving hydration, and strengthening the skin barrier, particularly in conditions such as rosacea and alcohol-related acneiform eruptions. Over-the-counter examples include The Ordinary Niacinamide 10% + Zinc 1% and Paula’s Choice 10% Niacinamide Booster [143]. Natural polyphenols, including resveratrol, offer additional benefits by inhibiting NF-κB signaling and ROS scavenging. Their use in dermocosmetics, such as Skinceuticals Resveratrol B E, provides potent nocturnal antioxidant defense, especially for patients exposed to oxidative insults from alcohol and ultraviolet radiation [142,144]. Zinc-based creams, like those containing zinc oxide or zinc pyrrolidone carboxylic acid, exhibit antimicrobial and anti-inflammatory effects, reduce irritative symptoms, and support epidermal healing. Their application is particularly relevant in alcohol-induced skin barrier disruption, where microbial colonization (e.g., *S. aureus*) exacerbates oxidative stress [64,145]. Emerging formulations also include topical GSH, carotenoids (e.g., lycopene, β-carotene), and selenium-enriched emulsions, although their dermal penetration and pharmacodynamics require further clinical validation [53,146].

Rutin, a natural plant-derived flavonoid, demonstrates promising properties as a co-active compound enhancing dermatological preparations’ photoprotective and antioxidant performance. Studies have shown that the addition of rutin to classical UVB filters, such as octocrylene, ethylhexyl methoxycinnamate, or 2-Ethylhexyl 4-(dimethylamino)benzoate (ethylhexyl dimethyl PABA), increases the radical scavenging activity by up to 75% compared to the use of UV filters alone. Furthermore, formulations containing rutin exhibited an extension of the critical wavelength, indicating improved UVA protection despite the absence of conventional UVA filters. Although rutin did not entirely prevent the photodegradation of UV filters, it significantly enhanced the overall photoprotection profile, especially in the UVA range, an essential factor in preventing photoaging and alcohol-induced oxidative stress. In vivo studies also confirmed good skin compatibility of rutin-enriched formulations, with preserved hydration, intact skin barrier function (as indicated by stable TEWL), and the absence of erythema. These findings highlight rutin’s potential as a valuable adjuvant in dermocosmetic products for skin compromised by ethanol metabolism and UV exposure [147].

Recent advances in nanotechnology have opened new perspectives for enhancing antioxidant therapy in alcohol-related dermatoses. Nanoparticle-based delivery systems such as solid lipid nanoparticles (SLNs), nanostructured lipid carriers (NLCs), liposomes, and polymeric nanocarriers significantly improve bioactive compounds’ penetration, stability, and controlled release. For example, SLNs and NLCs loaded with resveratrol, vitamin E, and epigallocatechin gallate have improved retention in the stratum corneum and deeper skin layers, offering a prolonged antioxidant effect and barrier repair function. These nanocarriers also enhance hydration, reduce transepidermal water loss, and modulate local inflammation factors, which are particularly relevant for skin compromised by chronic alcohol intake. Moreover, they allow targeting of skin appendages such as sebaceous glands and hair follicles, which may serve as reservoirs for therapeutic agents. Studies have shown that such formulations effectively treat conditions with shared oxidative and inflammatory pathways, including acne, psoriasis, and photoaging. Therefore, their integration into dermatocosmetics designed for alcohol-affected skin represents a logical, evidence-based innovation [148].

Despite the promise of antioxidant-based approaches, several limitations persist—particularly in long-term application. These include the instability of antioxidant compounds (e.g., vitamin C degradation), limited dermal penetration, and paradoxical pro-oxidative effects at high doses or prolonged exposure. To address these drawbacks, ongoing research explores multimodal strategies that combine antioxidants with complementary therapies. These include the use of prebiotics and probiotics to restore cutaneous microbial balance, adaptogens and immunomodulators (e.g., glycyrrhizin, panthenol), and specialized barrier-repair formulations enriched with ceramides, cholesterol, and fatty acids. Moreover, nanocarrier delivery systems—such as liposomes, solid lipid nanoparticles, and nanostructured lipid carriers—are being investigated to enhance the skin penetration, stability, and controlled release of active compounds. These adjunctive strategies may improve clinical outcomes and minimize side effects associated with long-term antioxidant monotherapy [133,149,150,151].

To enhance the clinical utility of antioxidant therapy in alcohol-related skin disorders, a stratified treatment algorithm should be proposed, tailored to the severity of oxidative damage and clinical presentation [152]. In patients with mild symptoms (e.g., dryness, scaling), dietary modification and supplementation with vitamins C and E may be sufficient. Moderate cases (e.g., erythema, acneiform eruptions) may benefit from topical formulations containing coenzyme Q10, resveratrol, niacinamide, or combinations thereof. Severe presentations (e.g., chronic inflammation, impaired wound healing) require a multimodal approach, combining antioxidant-rich nutrition, advanced topical agents, and delivery systems such as nanoparticles or liposomes. Biomarkers such as MDA, GSH, and SOD can guide monitoring [153,154]. Tentative efficacy thresholds—drawn from preclinical and clinical data—include a ≥20% reduction in MDA, ≥30% increase in SOD activity, and measurable improvement in TEWL and tissue regeneration [155,156,157,158]. These values are derived from the available preclinical and clinical literature and are intended as approximate reference points for therapeutic consideration. To emphasize, they should be interpreted within the context of broader clinical and nutritional assessment rather than as strict treatment thresholds.

In summary, the integration of antioxidant-rich dermatocosmetics into skin care regimens for individuals with alcohol use disorders holds great therapeutic potential. However, personalized approaches based on skin type, severity of alcohol-related damage, and nutritional deficiencies are essential. Furthermore, future studies should focus on bioavailability, optimal delivery systems (e.g., liposomes, nanocarriers), and long-term efficacy in clinical populations.

## 9. Conclusions

**Figure 1:**
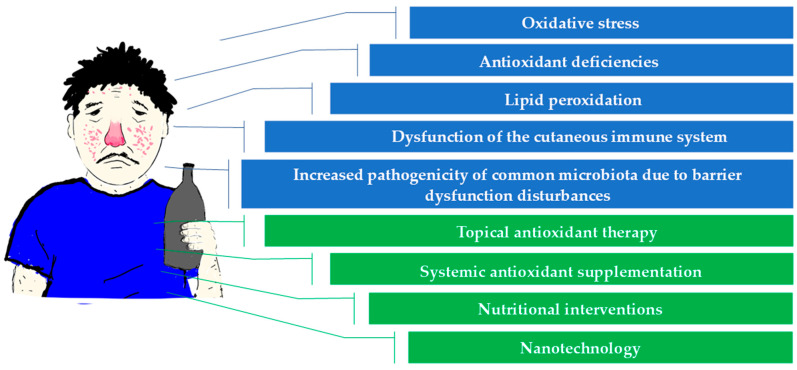
The impact of chronic alcohol consumption on skin health: mechanisms of damage and antioxidant-based therapeutic strategies.

Chronic alcohol consumption exerts multifaceted and deleterious effects on skin physiology, primarily through the induction of oxidative stress, disruption of epidermal barrier integrity, immune dysregulation, and altered redox homeostasis. Ethanol metabolism significantly elevates ROS levels, leading to lipid peroxidation, mitochondrial dysfunction, and DNA damage. These oxidative mechanisms contribute to impaired wound healing, increased susceptibility to infections, and the progression of various inflammatory and neoplastic skin conditions, including rosacea, psoriasis, atopic dermatitis, and skin cancers. Emerging evidence from in vitro and animal models confirms that ethanol impairs skin cell proliferation, collagen synthesis, and immune surveillance, particularly by reducing epidermal T and dendritic cells’ functionality. Alcohol-induced deficiencies in key antioxidants like GSH, vitamin C, vitamin E, and carotenoids impair the skin’s capacity to combat oxidative stress and preserve its structural integrity. Antioxidant supplementation offers therapeutic promise by restoring redox balance, modulating inflammatory pathways, and supporting epidermal repair mechanisms. However, clinical data remain limited, and further well-designed trials are needed to evaluate the efficacy, safety, and bioavailability of targeted antioxidant therapies in individuals with alcohol use disorders. Despite numerous studies on the effects of ethanol on the skin, there is still insufficient understanding of alcohol-specific molecular pathways. Further studies are required to elucidate the impact of ethanol and its metabolites, such as HER, on specific cellular damage mechanisms, including mitochondrial dysfunction and redox imbalance. Integrating antioxidant-based strategies with conventional dermatological and addiction treatments may represent a promising direction for personalized interventions to restore skin health in this vulnerable population. This summary is visually supported by Figure 1, which outlines the key mechanisms of alcohol-induced skin damage and highlights the therapeutic potential of antioxidant interventions.

## Figures and Tables

**Table 1:** Key antioxidants supporting skin health in alcohol-abusing individuals.

Antioxidant	Main Action	Dietary Sources	Clinical Evidence	Interactions and Notes	Refs.
Vitamin C	Collagen synthesis, neutralizes ROS, regenerates vitamin E	Citrus fruits, bell pepper, parsley	Improves skin elasticity, supports wound healing	High doses may increase oxidative stress in smokers	[51,56,57,73,74]
Vitamin E	Synergizes with vitamin C. Acts as the first line of defense against lipid peroxidation in cellular membranes	Nuts, seeds, vegetable oils	Reduces oxidative damage in alcoholics	May potentiate anticoagulant effects	[51,59,60,75,76,77,78,79]
GSH	Neutralizes ROS and RNS, cofactor for GPx and GST, supports glutathionylation, maintains redox homeostasis. The most crucial thiol-based redox buffer in the cell; regenerates vitamins C and E	Meat, cruciferous vegetables, avocado	Deficient in alcoholics, a key oxidative stress marker	GSH levels depend on cysteine intake; NAC enhances synthesis	[47,49,50,76,80,81,82]
CoQ_10_	Protects mitochondria, reduces lipid peroxidation, limits mitochondrial ROS production, supports electron transport	Fish, meat	Improves skin appearance, anti-aging effects	May affect blood pressure-lowering medications	[67,68,83]
Polyphenols (e.g., resveratrol)	Anti-inflammatory and antioxidant properties	Red wine, grapes, onions, tea	Reduces signs of photoaging and inflammation	Resveratrol may interact with anticoagulants	[84,85,86]
Selenium	Cofactor for GPx and TrxR, protects DNA, lipids, and proteins from oxidative damage	Fish, Brazil nuts, cereals	Low levels found in alcoholics, affects GPx activity	Excess intake may be toxic	[23,51,87,88,89]
β-carotene	Neutralizes singlet oxygen and peroxyl radicals, acts as provitamin A, may inhibit lipid peroxidation	Carrots, pumpkin, sweet potatoes	Decreases in skin after alcohol intake, reduces MED	High doses may increase lung cancer risk in smokers	[51,53,54,55,90,91,92]

Glutathione (GSH); glutathione peroxidase (GPx); glutathione S-transferase (GST); coenzyme Q_10_ (CoQ_10_); minimal erythema dose (MED); *N*-acetylcysteine (NAC); reactive nitrogen species (RNS); reactive oxygen species (ROS); thioredoxin reductase (TrxR).

## Abbreviations

The following abbreviations are used in this manuscript:

ADH	Alcohol dehydrogenase
ALDH	Aldehyde dehydrogenase
AUD	Alcohol use disorder
BCC	Basal cell carcinoma
CAT	Catalase
CDCs	Cutaneous dendritic cells
CoQ_10_	Coenzyme Q_10_
DETCs	Dendritic epidermal T cells
FAEEs	Fatty acid ethyl esters
HPV	Human papillomavirus
GPx	Glutathione peroxidase
GR	Glutathione reductase
GSH	Glutathione
GSSG	Oxidized glutathione, glutathione disulfide
GST	Glutathione S-transferase
HER	Hydroxyethyl radical
HNE	4-hydroxy-2-nonenal
ICD-11	International Classification of Diseases 11th Revision
IL-1β	Interleukin-1 beta
IL-6	Interleukin-6
IL-17	Interleukin-17
LPS	Lipopolysaccharide
MAPK	Mitogen-activated protein kinase
MDA	Malondialdehyde
NAC	*N*-acetylcysteine
MED	Minimal erythema dose
NAD^+^	Nicotinamide adenine dinucleotide
NADH	Reduced nicotinamide adenine dinucleotide
NADP^+^	Nicotinamide adenine dinucleotide phosphate
NADPH	Reduced nicotinamide adenine dinucleotide
NF-κB	Nuclear factor-κB
NLCs	Nanostructured lipid carriers
NOS	Nitric oxide synthase
NOX	NADPH oxidases
RNS	Reactive nitrogen species
ROS	Reactive oxygen species
SCC	Squamous cell carcinoma
SLNs	Solid lipid nanoparticles
SOD	Superoxide dismutase
TEWL	Transepidermal water loss
Th2-type	T helper type 2
TrxR	Thioredoxin reductase
TNF-α	Tumor necrosis factor alpha
UV	Ultraviolet
γδT17 cells	Dermal gamma delta T cells that produce interleukin-17
